# Developing a Prediction Model for 7-Year and 10-Year All-Cause Mortality Risk in Type 2 Diabetes Using a Hospital-Based Prospective Cohort Study

**DOI:** 10.3390/jcm10204779

**Published:** 2021-10-18

**Authors:** Sherry Yueh-Hsia Chiu, Ying Isabel Chen, Juifen Rachel Lu, Soh-Ching Ng, Chih-Hung Chen

**Affiliations:** 1 Department of Health Care Management, College of Management, Chang Gung University, Taoyuan 33302, Taiwan; sherrychiu@mail.cgu.edu.tw (S.Y.-H.C.); rachel@mail.cgu.edu.tw (J.R.L.); 2 Healthy Aging Research Center, Chang Gung University, Taoyuan 33302, Taiwan; 3 Division of Hepato-Gastroenterology, Department of Internal Medicine, Kaohsiung Chang Gung Memorial Hospital, Kaohsiung 83301, Taiwan; 4 Graduate Institute of Epidemiology and Preventive Medicine, College of Public Health, National Taiwan University, Taipei 10025, Taiwan; glamorous2238@gmail.com; 5 Graduate Institute of Management, College of Management, Chang Gung University, Taoyuan 33302, Taiwan; 6 Department of Radiation Oncology, Linkou Chang Gung Memorial Hospital, Linkou 33305, Taiwan; 7 Division of Endocrinology and Metabolism, Department of Internal Medicine, Keelung Chang Gung Memorial Hospital, Keelung 20401, Taiwan; angelang1127@gmail.com; 8 College of Medicine, Chang Gung University, Taoyuan 33302, Taiwan

**Keywords:** type 2 diabetes, all-cause mortality, prediction model, validation, prospective cohort, ROC curve

## Abstract

Leveraging easily accessible data from hospitals to identify high-risk mortality rates for clinical diabetes care adjustment is a convenient method for the future of precision healthcare. We aimed to develop risk prediction models for all-cause mortality based on 7-year and 10-year follow-ups for type 2 diabetes. A total of Taiwanese subjects aged ≥18 with outpatient data were ascertained during 2007–2013 and followed up to the end of 2016 using a hospital-based prospective cohort. Both traditional model selection with stepwise approach and LASSO method were conducted for parsimonious models’ selection and comparison. Multivariable Cox regression was performed for selected variables, and a time-dependent ROC curve with an integrated AUC and cumulative mortality by risk score levels was employed to evaluate the time-related predictive performance. The prediction model, which was composed of eight influential variables (age, sex, history of cancers, history of hypertension, antihyperlipidemic drug use, HbA1c level, creatinine level, and the LDL /HDL ratio), was the same for the 7-year and 10-year models. Harrell’s C-statistic was 0.7955 and 0.7775, and the integrated AUCs were 0.8136 and 0.8045 for the 7-year and 10-year models, respectively. The predictive performance of the AUCs was consistent with time. Our study developed and validated all-cause mortality prediction models with 7-year and 10-year follow-ups that were composed of the same contributing factors, though the model with 10-year follow-up had slightly greater risk coefficients. Both prediction models were consistent with time.

## 1. Introduction

Type 2 diabetes mellitus (T2DM) is a common chronic disease that imposes a significant financial burden on the health system. As T2DM is recognized as a systemic disease that often results in multiple complications, substantial direct and indirect medical expenditures related to routine care and complications throughout the lifetime of persons with T2DM [1] are likely to arise. Therefore, estimating the prospective economic cost from a population perspective is crucial to developing health policies. However, cost estimation is highly dependent on ethnicity, health care systems, culture, disease prevalence and progression, and mortality. Recently, Yang et al. organized a collaborative effort involving 22 prospective cohort studies in Asian countries with more than 1 million individuals to evaluate the risk of all-cause mortality in persons with T2DM. This study showed that the all-cause mortality risk in persons with T2DM was significantly higher by 1.89-fold than that in nondiabetic individuals; moreover, the risk varied by country and region and might well be influenced by socioeconomic status, health system, culture, etc. [2]. In the systematic review of prognostic indices for older adults, those explanatory variables were differentiated by settings (ex. community-dwelling patients, nursing home residents, etc.). Therefore, prognostic indices should be considered for heterogeneous populations to test accuracy [3]. The cohort study used for the prediction model, the Translating Research Into Action for Diabetes (TRIAD) study, started collecting data in 2000 and reported follow-up data at 4 years and 8 years: the significant factors for predicting all-cause mortality among T2DM patients were similar but with different coefficients [4,5]. However, more studies or cohorts are needed to examine whether this phenomenon can be applied in other countries. One would therefore conjecture that causes of death for persons with T2DM are multifaceted and that an all-cause mortality risk prediction model for persons with T2DM is crucial in assessing the economic impact of DM.

Taiwan launched the well-lauded National Health Insurance (NHI) program in 1995, and this program currently covers more than 99% of the population [6,7]. The Taiwan NHI provides Taiwan’s population of 23 million with comprehensive benefit coverages, which include prescription drugs, ambulatory visits to Western and Chinese medicine doctors and dentists, hospital emergency and inpatient services, home care, and hospice care. To enhance care quality for individuals with diabetes mellitus, the Bureau of NHI (now NHI Administration, NHIA) introduced a pay-for-performance (P4P) scheme for diabetes care in 2001 [8]. According to a report based on NHI claims data, the number of individuals with T2DM dramatically increased from 1.3 to 2.2 million between 2005 and 2014 [9] and reached 2.3 million in 2019 (11% prevalence rate) due to population aging, which may jeopardize the capacity of the health care system. Therefore, it is imperative to classify T2DM into different risk levels to enhance clinical management and aid health policy makers in impact assessment. The idea for this study is to develop the models based on clinical application in hospital (Supplementary Figure S1). The aim of this study was to develop, validate, and compare 7-year and 10-year risk prediction models of all-cause mortality in T2DM subjects based on a prospective cohort follow-up design.

## 2. Materials and Methods

### 2.1. Study Design, Population, and Data Source

We incorporated a database from one sizable regional hospital with 1089 beds, Chang Gung Memorial Hospital in Keelung (CGMH-K), located in Keelung City, northern Taiwan, which was founded by the Chang Gung Medical Foundation in 1985. The CGMH-K has provided an annual average of 175,000 outpatient visits and a fully engaged P4P program for diabetes care since 2007. Outpatient records from 1 Jan 2007 to 31 Dec 2013 were systematically retrieved from the hospital-based information management system, which was established in 1995 based on hospital administrative management and NHI reimbursement. Patients who were aged 18 or over and had at least one hospital admission or ≥3 outpatient visits recorded with the Classification (ICD) version ICD-9-CM code 250 within one year [10] were defined as having diabetes but excluding type 1 DM (coding 250.x1, 250.x3). A total of 18,202 T2DM subjects were recruited as our study population (Supplementary Figure S2).

### 2.2. Definitions for Comorbidity and Biomarkers

We also retrieved information on biochemical examinations (levels of HbA1c, cholesterol, HDL, creatinine, etc.), comorbidity history (hypertension, cancers, etc.), and drug treatments (antihypertension, antihyperlipidemia, etc.) from the hospital management system to generate individual factors/variables. Subjects who had three or more outpatient visits within one year with ICD-9-CM codes for hypertension or hyperlipidemia were defined as having a history of these diseases. Those for whom at least one visit was recorded within one year as cancers or peripheral vascular disease (PVD) (ICD-9-CM = 440, 441, 442, 443.1, 443.8, 443.9, 447.1, 785.4) were classified as having a history of cancer or PVD, respectively. The candidate predictors and definitions we used in this study have been described in Supplementary Table S1.

All biomarkers were assessed by the hospital centralized medical lab examination according to the standards of the College of American Pathologists (CAP) and recorded by the hospital electronic management system that was approved by the official central laboratory. In light of clinical laboratory criteria, patients whose biomarker results showed HbA1c < 7%, total cholesterol (TC) level < 200 mg/dL, triglyceride (TG) level < 150 mg/dL, low-density lipoprotein cholesterol (LDL ) level < 100 mg/dL, high-density lipoprotein (HDL ) level > 40 for males or >50 mg/dL for females, LDL /HDL ratio < 3.55 mg/dL for males and <3.22 mg/dL for females, and creatinine level 0.64–1.27 mg/dL for males and 0.44–1.13 mg/dL for females were defined as normal; otherwise, they were classified as abnormal subjects. For those with missing values for any of the biomarker variables, we used the missing-indicator method [11] to treat them as complete data for all analyses.

### 2.3. Study Observational End Points

We linked with the Taiwan National Mortality Registry System to ascertain the mortality information, including causes and date of death, using a unique number from the Health and Welfare Data Science Center (HWDC), which covers a nationwide official database and is governed by the Ministry of Health and Welfare, and followed up by the end of 2013 and 2016 for the 7-year and 10-year risk prediction models, respectively. This study protocol was reviewed and approved by the Institutional Review Board (IRB) of Chang Gung Memorial Hospital (issued numbers 103–3101B and 106–2459C).

### 2.4. Statistical Analysis

The individual follow-up person-years were calculated from the first date of those subjects diagnosed with T2DM and clinic visits between Jan. 2007 and Dec. 2013 to the date of death, which was treated as an event; otherwise, surviving patients were treated as censored. The censoring time points of Dec. 2013 and Dec. 2016 were applied for the 7-year and 10-year all-cause mortality model analyses, respectively. Time-to-event (death) analysis was employed to investigate the potential factors that affected all-cause mortality based on persons with T2DM in Taiwan. All statistical analyses were performed by SAS software, version 9.4 (SAS Institute Inc., Cary, North Carolina, USA). We also used SAS^®^ Viya^®^ 3.5 (SAS Visual Analytics) of Cloud Analytic Services (CAS) Library to perform LASSO (least absolute shrinkage and selection operator) method for model selection and best criterion value of model were selected based on SBC (Schwarz Bayesian criterion).

### 2.5. Model Selection and Development

The visualized graphical methods, plotting Schoenfeld residuals by time, were conducted to check the proportional hazards assumption (Supplementary Figure S3A,B). The multivariable Cox proportional hazards model was used to explore those factors and estimate the adjusted hazard ratio (aHR), which played a significant role in all-cause mortality for T2DM subjects, and was carried out using the stepwise approach with a *p*-value <0.05. Considering the number of variables included, the Akaike information criterion (AIC) was also applied for parsimonious model selection, and a lower AIC value was preferred. Besides the traditional model selection technique of stepwise approach, we also conducted the LASSO method that developed by Tibshirani [12] to compare the model selection. The plots for selection step of efficient sequence with standard coefficient and SBC criterion were generated demonstrated by selection procedures. The model with smaller SBC is better for selection.

### 2.6. Model Performance

As the continuous risk score generated by the prediction model, the receiver operating characteristic (ROC) curve was composed of sensitivity and specificity that were determined by different cutoff points. To evaluate the accuracy of our prediction models with long-term follow-up, Harrell’s C-statistic for time-to-event analysis was applied for predictive performance examination and employed the time-dependent area under the ROC curve (AUC) to check the predictive accuracy and consistency at different time points at which the 95% confidence interval (CI) of the AUC with the standard error (SE) computed by inverse-probability of censoring weighted (IPCW) was generated by 500 iterations. The integrated AUC for all time points was also adopted for evaluation [13,14,15]. 

### 2.7. Model Validation

The full samples were used to construct the risk prediction model based on multivariable Cox regression. First, based on the individual risk score, they were categorized into low- (<33.3%), intermediate- (33.3–66.6%), and high-risk (>66.6%) groups based on tertile grouping and demonstrated the cumulative mortality curves that were examined by simultaneous multiple comparisons with the Šidák correction adjustment [16]. For model internal validation, the samples were randomly divided into two groups of equal size. One half of the sample, the training data, was used as the estimation sample to obtain a set of parameter estimates based on the variables from the full sample. Then, the other half of the sample, the validation data, was used for validation, and the predicted mortality was compared with the actual observed mortality using a time-dependent ROC curve, AUC, and cumulative mortality curves (Supplementary Figure S6). Based on the LASSO approach for model selection, we also conducted random 50% dataset for each training and validation to validate those models with selected parameters. The efficient sequence for selection with SBC criterion were simultaneously demonstrated and compared with results of training and validation datasets.

## 3. Results

### 3.1. Characteristic of Study Subjects

The median follow-up time and number of deaths were 4.81 years (2779 deaths) and 6.75 years (4561 deaths) for the 7- and 10-year follow-ups, respectively (Supplementary Figure S2). A total of 18,202 T2DM subjects aged ≥18 years (mean age = 61.51, SD = 13.27) were recruited for this study, including 9065 females (49.8%) and 9137 males (50.2%). The distributions of age, year of study entry, and prevalence of diseases were similar between females and males. However, only total cholesterol levels, HDL levels, and the use of antihyperlipidemic drugs were slightly higher in females than in males (Supplementary Table S2). The all-cause mortality rates among individuals with T2DM were 3.50 and 3.71 per 100 for the 7-year and 10-year follow-ups, respectively. Higher mortality rates were observed for subjects with a history of cancer, PVD, hypertension, abnormal creatinine levels, and missing values on lipid profiles/biomarkers than in normal subjects or those with no history. Similar phenomena and trends were also observed at the 10-year follow-up (Table 1). The distribution of causes of mortality was demonstrated to have no significant difference between the 7-year and 10-year follow-ups. The major cause of death was cancer (23–24%) (Supplementary Table S3).

### 3.2. Factors and Coefficients of Prediction Models for All-Cause Mortality

**Figure 1:**
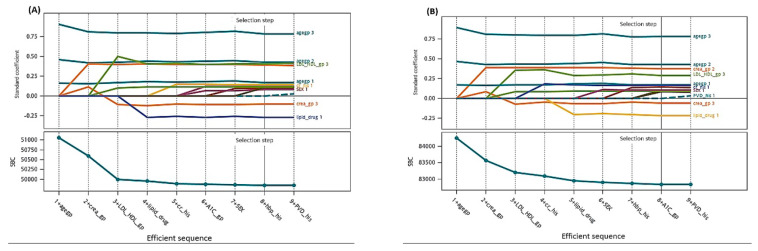
The coefficient progression for time with selection steps for model selection using LASSO method: (**A**) 7-year and (**B**) 10-year model.

Before the Cox regression analysis, our data did not violate the assumption of proportional hazards according to the graphic method with Schoenfeld residuals over time. Taking HbA1c as an example, the three categories (normal, abnormal, and missing) were parallel to each other and independent of time (Supplementary Figure S3A for 7 years and S3B for 10 years). First, parsimonious multivariable Cox regression models were developed by stepwise selection and AIC criteria (Supplementary Table S4). The second and fourth columns in Table 2 present the adjusted HR prediction model for the 7-year and 10-year follow-up data. The variables that reached statistical significance included male sex, history of cancer, history of hypertension, abnormal HbA1c, high creatinine levels, and LDL /HDL ratio with adjusted HRs of 1.21, 1.40, 1.30, 1.28, 2.50, and 1.29, respectively. For patients aged <50 y/o, the adjusted HRs were 1.48, 2.69, and 5.64 for those aged 50–59, 60–69, and ≥70, respectively. However, for those who use antihyperlipidemic drugs, the adjusted HR shows a protective effect on all-cause mortality of 0.58. A similar adjusted HR trait was also present at the 10-year follow-up, but with a slight increase (Table 2). In addition to age, abnormal creatinine levels, as a parameter of kidney function, demonstrate a higher risk of all-cause mortality for persons with T2DM. Furthermore, based on the LASSO method for model selection using SBC criterion, those selected variables for final models for 7-year and 10-year were same as stepwise approach (Figure 1, (A) 7-year, (B) 10-year). Both standard coefficients for variables and SBC criterion can demonstrate the efficient sequence of variables on all-cause mortality. Those SBC for selection steps were indicated by best criterion value (with *). The selected models were with same selected variables for both 7-year and 10-year models, respectively, but the effect order of steps was slightly different (Supplementary Table S5). The results of LASSO method demonstrated the similar trait for those selected variables (Supplementary Table S6).

The final prediction model for all-cause mortality was developed based on the model selection for 7-year and 10-year follow-ups. In addition to the adjusted HRs presenting the risk of mortality, the coefficients of the final parsimonious models are also provided in Table 2 for individual all-cause mortality risk prediction. The all-cause mortality risk scores can be calculated as follows:

- 7-year all-cause mortality risk score for individuals with type 2 diabetes
  = 0.3941 × aged 50–59 (if yes = 1) + 0.9882 × aged 60–69 (if yes = 1) + 1.7294 × aged ≥ 70 (if yes = 1)
  + 0.1867 × sex (if male = 1)
  + 0.3364 × history of cancer (if yes = 1)
  + 0.2615 × history of hypertension (if yes = 1)
  − 0.5407 × use of antihyperlipidemic drugs (if yes = 1)
  + 0.2440 × HbA1c (if abnormal = 1) + 0.2154 × HbA1c (if missing = 1)
  + 0.9154 × creatinine (if abnormal = 1) − 0.3088 × creatinine (if missing = 1)
  + 0.2569 × LDL /HDL ratio (if abnormal = 1) + 0.9216 × LDL /HDL ratio (if missing = 1)
- 10-year all-cause mortality risk score for individuals with type 2 diabetes
  = 0.3910 × aged 50–59 (if yes = 1) + 0.9908 × aged 60–69 (if yes = 1) +1.7198 × aged ≥ 70 (if yes = 1)
  + 0.2163 × sex (if male = 1)
  + 0.3860 × history of cancer (if yes = 1)
  + 0.3439 × history of hypertension (if yes = 1)
  − 0.4290 × use of antihyperlipidemic drugs (if yes = 1)
  + 0.2131 × HbA1c (if abnormal = 1) + 0.2126 × HbA1c (if missing =1)
  + 0.8793 × creatinine (if abnormal = 1) − 0.1934 × creatinine (if missing = 1)
  + 0.2056 × LDL /HDL ratio (if abnormal = 1) − 0.6472 × LDL /HDL ratio (if missing=1)

Example for score calculation:

Male with baseline condition and 10-year follow-up: 65 years old, HbA1c = 7.5 (abnormal), creatinine level = 1.2 (normal), LDL /HDL ratio = 4.06 (abnormal), a history of cancer and hypertension, use of antihyperlipidemic drugs


Score = 0.9908 + 0.2163 + 0.3860 + 0.3439 − 0.4290 + 0.2131 + 0.2056 = 1.9267

### 3.3. Performance of Prediction Models for All-Cause Mortality

**Figure 2:**
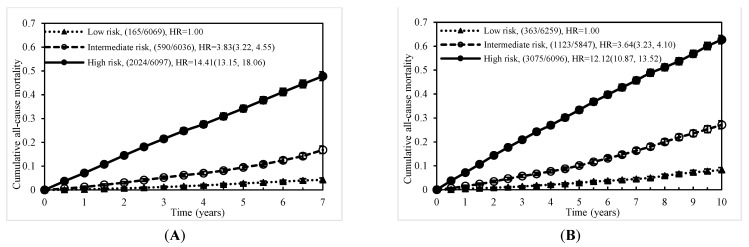
Cumulative mortality due to all causes by risk score level: (**A**) at 7 years and (**B**) at 10 years.

**Figure 3:**
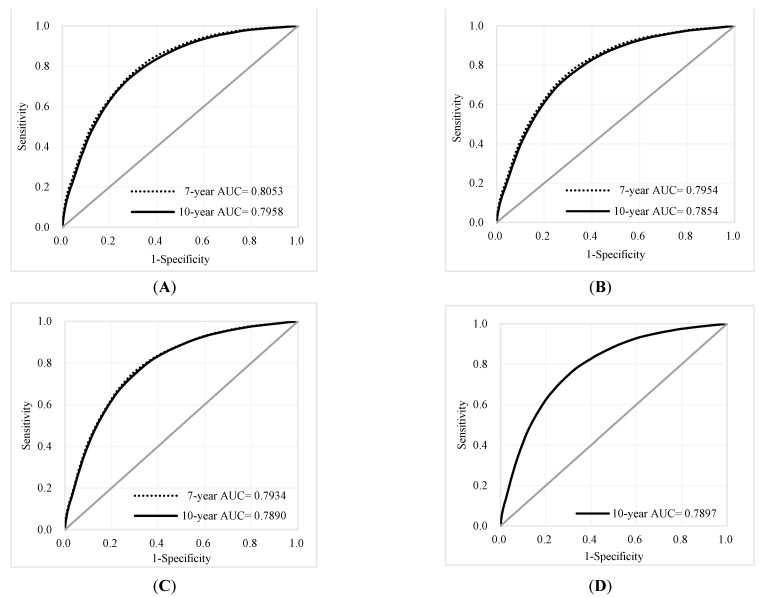
ROC curves and AUCs at different time points for the 7-year and 10-year follow-ups: (**A**) 2nd year; (**B**) 4th year (**C**) 6th year, (**D**) 8th year for 10-year only.

**Figure 4:**
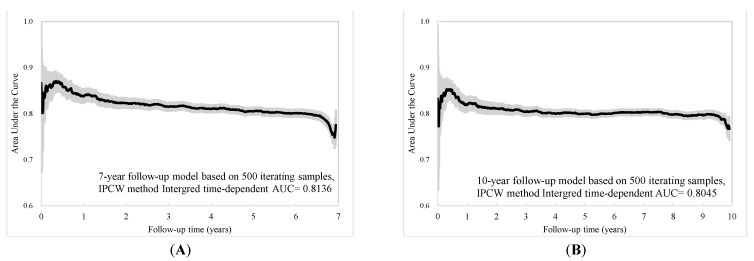
Time-dependent AUC with 95% confidence interval based on (**A**) 7-year and (**B**) 10-year follow-up.

The individual risk score was generated based on the coefficients of the final Cox regression and the low-, intermediate-, and high-risk groups. The risk prediction by using cumulative all-cause mortality was successfully discriminated at 7 years and 10 years (Figure 2A,B), and both *p*-values of the log-rank test were <0.0001. To evaluate the concordance of the prediction model for the time to death based on the final models that we developed, considering the time-dependent dynamic event, Harrell’s C-statistic was 0.7955 (95% CI: 0.7873, 0.8037) and 0.7775 (95% CI: 0.7708, 0.7842) for the 7-year and 10-year models, respectively (Table 2). The time-varying AUCs at the 2nd, 4th, and 6th years were 0.8053, 0.7954, and 0.7934 for the 7-year follow-up and 0.7958, 0.7854, 0.7890, and 0.7897 (8th year) for the 10-year follow-up, respectively. These AUCs did not show significant differences at different follow-up times (Figure 3A–D). Furthermore, considering the predictive performance of the AUC with 95% CI at continuous times, the IPCW method with 500-iterating samples demonstrated a slightly high AUC within one year, and AUCs were consistent with the follow-up time regardless of the different time points. The same pattern was shown in the 7-year and 10-year models (Figure 4A,B).

### 3.4. Validation of Prediction Models for All-Cause Mortality

First, using the random half of dataset (training), the model selection based on SBC and best criterion value, the results of variables selected were the same as our final model. The SBC for both 7-year and 10-year were shown on Supplementary Table S7. Second, based on those 8 parameters of selected models, the random 50% cross-validation showed patterns of standard coefficient and coefficient progression step were similar (Figure S4A for 7-year and S5A for 10-year). Comparing log-likelihood of training with validation datasets, they were close to each other for the selection step (Figure S4B for 7-year and Figure S5B for 10-year). On the other hand, the time-dependent AUC based on cross-validation with 9101 and 9101 subjects for the training and validation datasets, respectively, was employed to validate the predictive performance, and the schema is shown in Supplementary Figure S6. The distributions of variables between the training and validation data were not significantly different (Supplementary Table S8). Second, the cumulative all-cause mortality curves showed that the predicted and observed data were very close regardless of whether the 7-year or 10-year follow-up data were assessed (Supplementary Figure S7A,B). For the performance validation of prediction model, the ROC curves and AUCs for the 2nd-, 4th-, 6th-, and 10th-year time points also demonstrated no significant difference (Supplementary Figure S8A–C and Supplementary Figure S9A–D).

## 4. Discussion

The CGMH-K is the largest hospital in Keelung, northern Taiwan, and cares for one-third of the people with T2DM in Keelung City, according to NHI statistics. Some studies of all-cause mortality prediction from Western countries have been reported, but few have been based on Taiwan, in which the national health insurance covers more than 99% of the population. Therefore, our study described the development of a prediction model for all-cause mortality based on data from individuals with T2DM. The predictive performance of the C-statistic was 0.7955 and 0.7775, and the integrated time-dependent AUC reached 0.8136 and 0.8045 for the 7-year and 10-year follow-up, respectively. The performance was also consistent at different time points; moreover, the cross-validation demonstrated a good fit for different risk levels. Compared with our prediction models for all-cause mortality, the performance of the C-statistic was 0.80 in a multiethnic study in New Zealand [17], 0.77 (male) and 0.78 (female) in a Chinese study in Hong Kong [18], and 0.81 in a cohort study in Italy [19,20]. Regardless of ethnicity, these results were similar, and the prediction performance was slightly higher for females than for males. Our C-statistics for performance by sex are presented in Supplementary Table S9.

Epidemiological studies and the biological mechanism of inflammation in diabetes have demonstrated that diabetes is an independent risk factor for the incidence of specific cancers and increases the risk of all-cause mortality and poor prognosis [21]. On the other hand, according to the vital statistics reported by the Taiwan Ministry of Health and Welfare, overall cancer mortality has been the leading cause of mortality in Taiwan for over three decades. Therefore, the development of a risk prediction model for diabetes could not omit cancer status from the estimation, while the impact of cancer on health is well recognized. As shown in our results, a history of cancer was associated with a 1.47-fold (95% CI: 1.38, 1.57) increased risk of all-cause mortality. In light of a previous all-cause mortality prediction model for T2DM that was constructed based on the Hong Kong Diabetes Registry, a history of cancer presented the highest risk as a significant prediction factor [22]. However, the prediction model was based on a Hong Kong Chinese population excluding subjects who were diagnosed with cardiovascular disease (CVD) or cancers at baseline; more importantly, there was a high prevalence rate of cancers in our study (23.4%, Supplementary Table S2), and cancer and CVD were the top two leading causes of death in Taiwan (Supplementary Table S3) and other countries. This would underestimate the impact of diabetes on the outcome spectrum, especially on all-cause mortality. Though Hong Kong and Taiwan have similar ethnic Chinese populations (but different cultural and health care systems), the overall mortality rate in Hong Kong was 4.67% (male: 5.81%, female: 3.68%) [17], which was higher than that in the Taiwanese study (overall: 3.50%, male: 3.66%, female: 3.34%). Further study is needed to explore the factors/reasons contributing to the difference in mortality.

CVD is ranked as the leading cause of death and an important health care issue worldwide, but a high blood cholesterol level is a major determinant of CVD. Cholesterol-lowering drugs, such as statins, were developed in the 1990s and have also been issued for clinical care and covered by National Health Insurance in Taiwan since 2003. Our results showed that after adjustment for other significant factors, compared with no use of hyperlipidemia drugs, the use of antihyperlipidemic drugs significantly reduced all-cause mortality. In 2013, a meta-analysis based on several trials demonstrated the significant 14% reduction in all-cause mortality [23], and a meta-analysis based on statin trials with long-term follow-up (posttrial) found a 10% all-cause mortality reduction [24]. In 2017, a study with a 5-year follow-up based on people from Hong Kong with T2DM reported that statin use significantly reduced CVD risk and all-cause mortality (adjusted HR = 0.487) [25]. In 2018, Chen et al. conducted a retrospective cohort study based on hospital outpatients with T2DM in central Taiwan to evaluate the effect of statin use on all-cause mortality, and the results also demonstrated a significant reduction benefit [26]. It is obvious that the use of antihyperlipidemic drugs can make a significant contribution to reducing all-cause mortality, and we could not omit this factor from the prediction model for all-cause mortality, especially for subjects from recent healthcare databases.

In 2019, Li et al. reported the annual all-cause mortality in persons with T2DM between 2005–2014 using the Taiwan NHI nationwide-scale database based on the same criteria as our study using the annual deaths divided by the prevalence of T2DM among individuals who were alive on 1 January of each year. The annual mortality rates were 3.24% for all persons with T2DM, 2.93% for females, and 3.54% for males [9]. Our study demonstrated that the all-cause mortality rates were 3.50%, 3.34%, and 3.66% for all individuals, females, and males with T2DM, respectively, based on a 7-year follow-up. Compared with Li et al. [9], who employed a one-year follow-up, the slightly higher mortality rate in our study can be attributed to a longer-term follow-up. Moreover, the NHI database constructed from administrative claims data using ICD diagnosis codes does not include important biomarkers, such as levels of TG, HDL, HbA1c, creatinine, etc. Moreover, our study exploits a hospital-based prospective cohort with rich laboratory biomarker information, which is crucial to complement the development of a risk prediction model.

The study by Li et al., which linked the Taiwan National Diabetes Care Management Program (NDCMP) with the Health Insurance Research Database using the same criteria as our study to identify T2DM subjects between 2001–2004 and calculated in-hospital mortality by follow-up until the end of 2011, found similar results as our study [27]. An abnormal creatinine level was identified as a highly significant risk predictor for mortality, and the prediction AUCs for in-hospital mortality at 5 and 8 years were 0.770 and 0.756, respectively. Compared with our study, the model reported by Li et al. [26] restricted the outcome to in-hospital deaths only; consequently, patients who died outside the hospital were not included, whereas our study linked the individual data with a nationwide death registry to identify all deaths. In addition, cancer history was not included in Li et al.’s model development. The performance of the prediction model might be enhanced if these two issues were addressed.

Missing values for important variables, such as HbA1c, LDL, and HDL levels, which suggests that persons with T2DM have low compliance or may miss regular follow-up visits (Table 1), is also an issue to address in our study. We hence adopted the missing-indicator method to include those participants for complete data analysis, as it may capture health awareness or compliance into consideration.

Some research limitations bear mentioning in our study. The first is data limitation. Although our study sample was constructed with persons with T2DM from only one sizable regional hospital, CGMH-K, this hospital covers more than one-third of individuals with T2DM care in the northern City-Keelung. Hence, our study sample is still representative of the population. Our data also lack health-related behavioral factors, such as exercise, alcohol consumption, and cigarette smoking, which are usually unavailable in hospital-based datasets.

Second, model validation using an external population, such as persons with T2DM at hospitals of the same level in different counties, would be ideal but unfortunately not obtainable at the time of the study due to time and resource constraints. However, the internal validation results seem satisfactory.

Third, quality of care, sociodemographic characteristics, and individuals’ levels of health awareness might vary by region in Taiwan; therefore, the prediction model might be slightly different among cities and counties. However, the rigorous approaches adopted in the development of our risk prediction model, including variable ascertainment and external validation, can still provide good references for other hospitals interested in building risk prediction models for clinical and research applications.

## 5. Conclusions

Our study developed and validated an all-cause mortality prediction model based on Taiwanese hospital-based diabetes with 7-year and 10-year follow-ups. The methods and risk prediction parameters can be applied to identify high-risk mortality in hospital clinical care and to further assess the net value of treatment options in economic evaluation.

## Figures and Tables

**Table 1:** All-cause mortality rates of persons with type 2 diabetes mellitus by characteristics and risk factors.

Variables	7-Year Follow-Up			10-Year Follow-Up		
	No. Deaths	Person Years	Mortality Rate (per 100) (95% CI)	No. Deaths	Person Years	Mortality Rate (per 100) (95% CI)
Overall	2779	79,427.1	3.50 (2.20, 4.80)	4561	12,2929.5	3.71 (2.63, 4.79)
Age at entry						
<50 y/o	169	16,277.6	1.04 (0.00, 2.61)	307	26,328.9	1.17 (0.00, 2.47)
50–59 y/o	322	21,426.9	1.50 (0.00, 3.14)	588	34,384.4	1.71 (0.33, 3.09)
60–69 y/o	601	19,729.5	3.05 (0.61, 5.49)	1055	30,701.2	3.44 (1.37, 5.51)
≥70 y/o	1687	21,993.1	7.67 (4.01, 11.33)	2611	31,515.0	8.28 (5.10, 11.46)
Sex						
Female	1337	40,035.8	3.34 (1.55, 5.13)	2179	61,942.9	3.52 (2.04, 5.00)
Male	1442	39,391.3	3.66 (1.77, 5.55)	2382	60,986.6	3.91 (2.34, 5.48)
History of cancer						
No	1867	60,762.9	3.07 (1.68, 4.46)	3075	95,153.6	3.23 (2.09, 4.37)
Yes	912	18,664.2	4.89 (1.72, 8.06)	1486	27,775.8	5.35 (2.63, 8.07)
History of PVD						
No	2635	76,777.0	3.43 (2.12, 4.74)	4326	11,9076.3	3.63 (2.55, 4.71)
Yes	144	2650.1	5.43 (0.0, 14.31)	235	3853.2	6.10 (0.00, 13.90)
History of hypertension						
No	381	16,555.4	2.30 (0.0, 4.61)	581	27,295.1	2.13 (0.40, 3.86)
Yes	2398	62,871.7	3.81 (2.28, 5.34)	3980	95,634.3	4.16 (2.87, 5.45)
Use of antihypertensive drugs						
No	648	23,583.9	2.75 (0.63, 4.87)	967	38,361.7	2.52 (0.93, 4.11)
Yes	2131	55,843.2	3.82 (2.20, 5.44)	3594	84,567.8	4.25 (2.86, 5.64)
History of hyperlipidemia						
No	1478	23,427.9	6.31 (3.09, 9.53)	2124	36,215.8	5.86 (3.37, 8.35)
Yes	1301	55,999.2	2.32 (1.06, 3.58)	2437	86,713.7	2.81 (1.69, 3.93)
Use of antihyperlipidemic drugs						
No	1685	33,496.2	5.03 (2.63, 7.43)	2517	51,989.5	4.84 (2.95, 6.73)
Yes	1094	45,930.9	2.38 (0.97, 3.79)	2044	70,940.0	2.88 (1.63, 4.13)
HbA1c						
Normal (<7)	1034	33,460.8	3.09 (1.21, 4.97)	1754	51,138.8	3.43 (1.82, 5.04)
Abnormal (≧7)	1081	33,826.6	3.20 (1.29, 5.11)	1838	51,850.1	3.54 (1.92, 5.16)
Missing	664	12,139.8	5.47 (1.31, 9.63)	969	19,940.6	4.86 (1.80, 7.92)
Creatinine						
Normal	1191	54,274.7	2.19 (0.94, 3.44)	2084	84,441.7	2.47 (1.41, 3.53)
Abnormal	1379	18,294.5	7.54 (3.56, 11.52)	2107	26,179.9	8.05 (4.61, 11.49)
Missing	209	68,57.9	3.05 (0.0, 7.18)	370	12,307.9	3.01 (0.0, 6.07)
Total cholesterol						
Normal (<200)	1460	44,192.5	3.30 (1.61, 4.99)	2406	67,844.4	3.55 (2.13, 4.97)
Abnormal (≧200)	606	25,712.4	2.36 (0.48, 4.24)	1140	39,098.5	2.92 (1.23, 4.61)
Missing	713	9522.2	7.49 (1.99, 12.99)	1015	15,986.6	6.35 (2.44, 10.26)
Triglyceride						
Normal (<150)	1392	44,884.8	3.10 (1.47, 4.73)	2321	67,964.7	3.42 (2.03, 4.81)
Abnormal (≧150)	626	24,692.3	2.54 (0.55, 4.53)	1172	38,567.3	3.04 (1.30, 4.78)
Missing	761	9850.0	7.73 (2.24, 13.22)	1068	16,397.4	6.51 (2.60, 10.42)
LDL						
Normal (<100)	720	22,242.0	3.24 (0.88, 5.60)	1253	34,843.8	3.60 (1.61, 5.59)
Abnormal (≧100)	1143	44,533.7	2.57 (1.08, 4.06)	2026	67,480.9	3.00 (1.69, 4.31)
Missing	916	12,651.3	7.24 (2.55, 11.93)	1282	20,604.8	6.22 (2.81, 9.63)
HDL						
Normal	424	17,769.8	2.39 (0.12, 4.66)	782	28,603.1	2.73 (0.81, 4.65)
Abnormal	1430	49,105.7	2.91 (1.40, 4.42)	2483	73,860.2	3.36 (2.04, 4.68)
Missing	925	12,551.7	7.37 (2.62, 12.12)	1296	20,466.2	6.33 (2.88, 9.78)
LDL /HDL ratio						
Normal	928	37,921.2	2.45 (0.88, 4.02)	1737	58,961.9	2.95 (1.56, 4.34)
Abnormal	826	26,064.1	3.17 (1.01, 5.33)	1368	37,433.1	3.65 (1.71, 5.59)
Missing	1025	15,441.8	6.64 (2.58, 10.70)	1456	26,534.5	5.49 (2.67, 8.31)

PVD: peripheral vascular disease; normal creatinine level: male <1.27, female <1.13 mg/dL; Abnormal HDL level: male <40, female <50 mg/dL; abnormal LDL /HDL ratio: male >3.55, female >3.22.

**Table 2:** Results from univariate and multivariable Cox regressions predicting all-cause mortality. Among persons with type 2 diabetes mellitus.

Variable	10-Year Model							
	Univariate		Multivariable		Univariate		Multivariable	
	β	HR (95% CI)	β	aHR (95% CI)	β	HR (95% CI)	β	aHR (95% CI)
Age at entry								
50–59 vs. <50 y/o	0.3698	1.45 (1.20, 1.74)	0.3941	1.48 (1.23, 1.79)	0.3838	1.47 (1.28, 1.69)	0.3910	1.48 (1.29, 1.70)
60–69 vs. <50 y/o	1.0729	2.92 (2.47, 3.47)	0.9882	2.69 (2.26, 3.19)	1.0815	2.95 (2.60, 3.35)	0.9908	2.69 (2.37, 3.06)
≥70 vs. <50 y/o	1.9937	7.34 (6.27, 8.60)	1.7294	5.64 (4.79, 6.64)	1.9628	7.12 (6.33, 8.01)	1.7198	5.58 (4.94, 6.31)
Sex								
Male vs. Female	0.0917	1.10 (1.02, 1.18)	0.1867	1.21 (1.12, 1.30)	0.1059	1.11 (1.05, 1.18)	0.2163	1.24 (1.17, 1.32)
History of cancer								
Yes vs. No	0.4638	1.59 (1.47, 1.72)	0.3364	1.40 (1.29, 1.52)	0.5027	1.65 (1.55, 1.76)	0.3860	1.47 (1.38, 1.57)
History of hypertension								
Yes vs. No	0.5123	1.67 (1.50, 1.86)	0.2615	1.30 (1.15, 1.46)	0.6702	1.96 (1.79, 2.13)	0.3439	1.41 (1.28, 1.55)
Use of antihyperlipidemic drugs								
Yes vs. No	−0.7397	0.48 (0.44, 0.52)	−0.5407	0.58 (0.53, 0.64)	−0.5182	0.60 (0.56, 0.63)	−0.4290	0.65 (0.61, 0.70)
HbA1c								
≧7 vs. <7	0.0307	1.03 (0.95, 1.12)	0.2440	1.28 (1.17, 1.39)	0.0316	1.03 (0.97, 1.10)	0.2131	1.24 (1.16, 1.32)
Missing vs. <7	0.5524	1.74 (1.58, 1.92)	0.2154	1.24 (1.10, 1.40)	0.3463	1.41 (1.31, 1.53)	0.2126	1.24 (1.12, 1.37)
Creatinine								
Abnormal vs. normal	1.2279	3.41 (3.16, 3.74)	0.9154	2.50 (2.31, 2.71)	1.1820	3.26 (3.07, 3.47)	0.8793	2.41 (2.26, 2.56)
Missing vs. normal	0.3032	1.35 (1.17, 1.57)	−0.3088	0.73 (0.62, 0.87)	0.2035	1.23 (1.10, 1.37)	−0.1934	0.82 (0.73, 0.94)
LDL /HDL ratio								
Abnormal vs. normal	0.2725	1.31 (1.20, 1.44)	0.2569	1.29 (1.18, 1.42)	0.2177	1.24 (1.16, 1.34)	0.2056	1.23 (1.14, 1.32)
Missing vs. normal	0.9760	2.65 (2.43, 2.90)	0.9216	2.51 (2.26, 2.80)	0.6260	1.87 (1.74, 2.01)	0.6472	1.91 (1.75, 2.08)
Harrell’s C-statistic			0.7955	(0.7873, 0.8037)			0.7775	(0.7708, 0.7842)
Integrated time-dependent AUC			0.8169				0.8085	

Normal creatinine level: male < 1.27, female: < 1.13 mg/dL; abnormal HDL level: male < 40, female < 50 mg/dL. Abnormal LDL /HDL ratio: male > 3.55, female > 3.22; aHR: adjusted hazard ratio.

## Data Availability

The data presented in this study are available on request from the corresponding author. The data are not publicly available due to committee process.

## Supplementary Materials

The following are available online at www.mdpi.com/article/10.3390/jcm10204779/s1, Figure S1: Proposed study scenario and application; Figure S2: Flow diagram for study subjects; Figure S3 (A): Proportional hazards assumption checking for the 7-year follow- up; Figure S3 (B): Proportional hazards assumption checking for the 10-year follow- up; Figure S4: The (A) Coefficient Progression with selection steps and (B) efficient sequence of cross-validation (7-year model); Figure S5: The (A) Coefficient Progression with selection steps and (B) efficient sequence of cross-validation (10-year model); Figure S6: Flowchart for model cross-validation; Figure S7 (A): Cumulative all-cause mortality for the 7-year follow-up based on 9101 validation subjects; Figure S7 (B): Cumulative all-cause mortality for the 10-year follow-up based on 9101 validation subjects; Figure S8 (A): ROC and AUC for cross-validation at the 2-year time point based on the 7-year follow-up; Figure S8 (B): ROC and AUC for cross-validation at the 4-year time point based on the 7-year follow-up; Figure S8 (C): ROC and AUC for cross-validation at the 6-year time point based on the 7-year follow-up. Figure S9 (A): ROC and AUC for cross-validation at the 2-year time point based on the 10-year follow-up; Figure S9 (B): ROC and AUC for cross-validation at the 4-year time point based on the 10-year follow-up; Figure S9 (C): ROC and AUC for cross-validation at the 6-year time point based on the 10-year follow-up; Table S1: Distribution of patient characteristics and risk factors by sex; Figure S9 (D): ROC and AUC for cross-validation at the 8-year time point based on the 10-year follow-up; Table S1: Description of variables, candidate predictors and definition in this study; Table S2: Distribution of patient characteristics and risk factors by sex; Table S3: Numbers and causes of deaths by the 7-year and 10-year follow-ups; Table S4: AIC for model selection; Table S5: Model selection using SBC with best criterion value for 7-year and 10-year model; Table S6: The results of regression coefficient for 7-year and 10-year using Lasso method; Table S7: Model selection based on training data using SBC with best criterion value for 7-year and 10-year model; Table S8: Distribution of patient characteristics and risk factors by training and validation data; Table S9: Harrell’s C statistic by sex based on 7-year and 10-year follow-ups.
