# Supplementary material for: Developing a Prediction Model for 7-Year and 10-Year All-Cause Mortality Risk in Type 2 Diabetes Using a Hospital-Based Prospective Cohort Study
Source: J Clin Med. 2021 Oct 18;10(20):4779. doi: 10.3390/jcm10204779 (PMC8537078; doi:10.3390/jcm10204779)
Supplement: Supplementary file 1 [file jcm-10-04779-s001.zip › 2021-08-25-JCM_revision 2_Supplementary Figures.pdf]

Developing a Prediction Model for 7-year and 10-year All-cause Mortality Risk in Type 2 Diabetes Using a Hospital-based Prospective Cohort Study

Sherry Yueh-Hsia Chiu[1,2], Ying Isabel Chen[3], Jui-fen Rachel Lu[4,5], Soh-Ching Ng[6], Chih-Hung Chen[6*]

1. Department of Health Care Management, College of Management; and Healthy Aging Research Center, Chang Gung University, Taiwan; sherrychiu@mail.cgu.edu.tw (S.Y.-H. Chiu)
2. Division of Hepato-gastroenterology, Department of Internal Medicine, Kaohsiung Chang Gung Memorial Hospital, Taiwan
3. Graduate Institute of Epidemiology and Preventive Medicine, College of Public Health, National Taiwan University, Taipei, Taiwan; glamorous2238@gmail.com (Y.I. Chen)
4. Graduate Institute of Business and Management and Department of Health Care Management, College of Management, Taoyuan, Chang Gung University, Taiwan; rachel@mail.cgu.edu.tw (J.-f. R. Lu)
5. Department of Radiation Oncology, Linkou Chang Gung Memorial Hospital, Linkou, Taiwan
6. Division of Endocrinology and Metabolism, Department of Internal Medicine, Keelung Chang Gung Memorial Hospital, Keelung; Chang Gung University, Taiwan; angelang1127@gmail.com (S.-C. Ng);

*Correspondence: yh1008@cgmh.org.tw (C.-H. Chen)

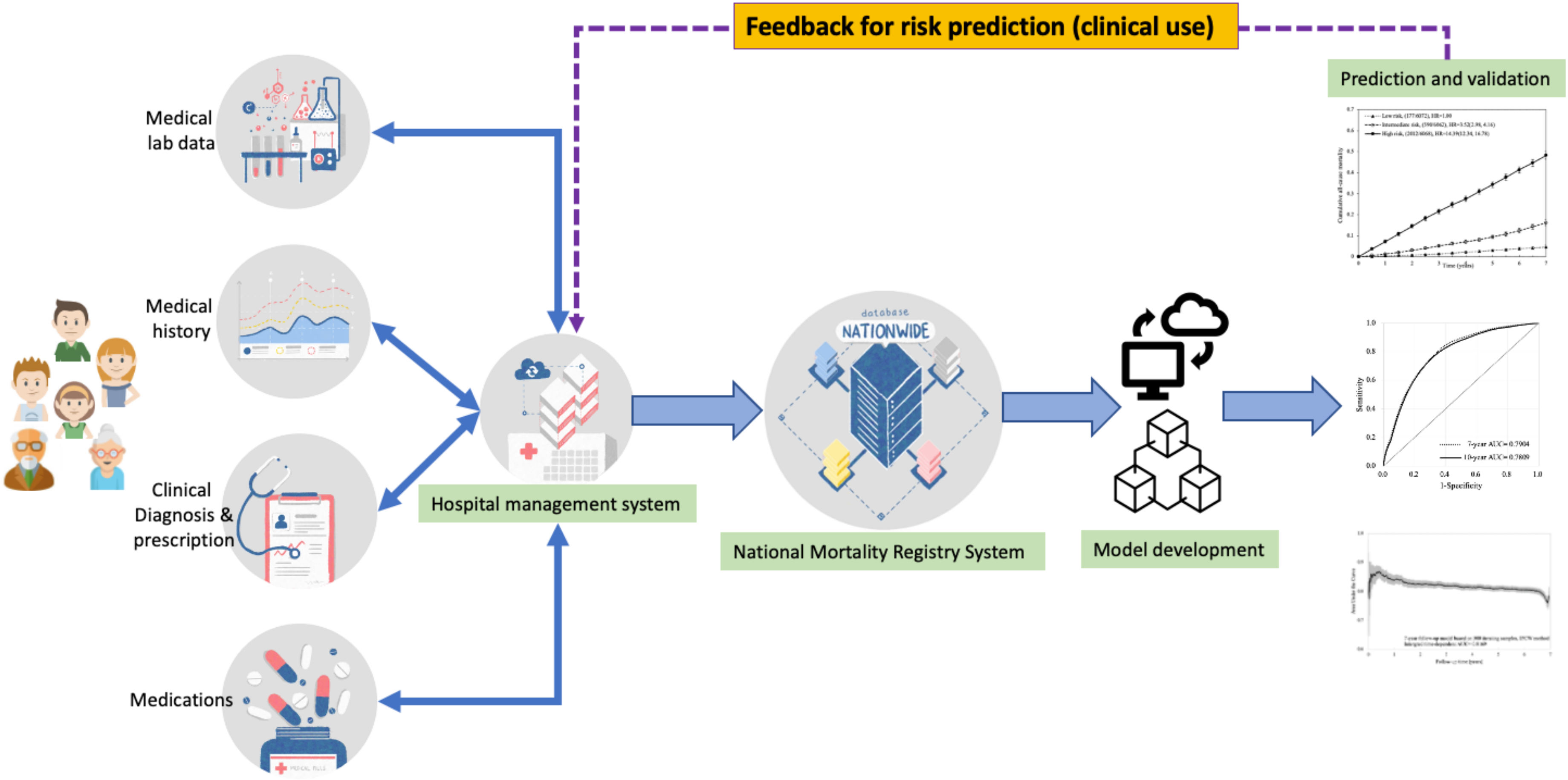

Figure S1. Proposed study scenario and application

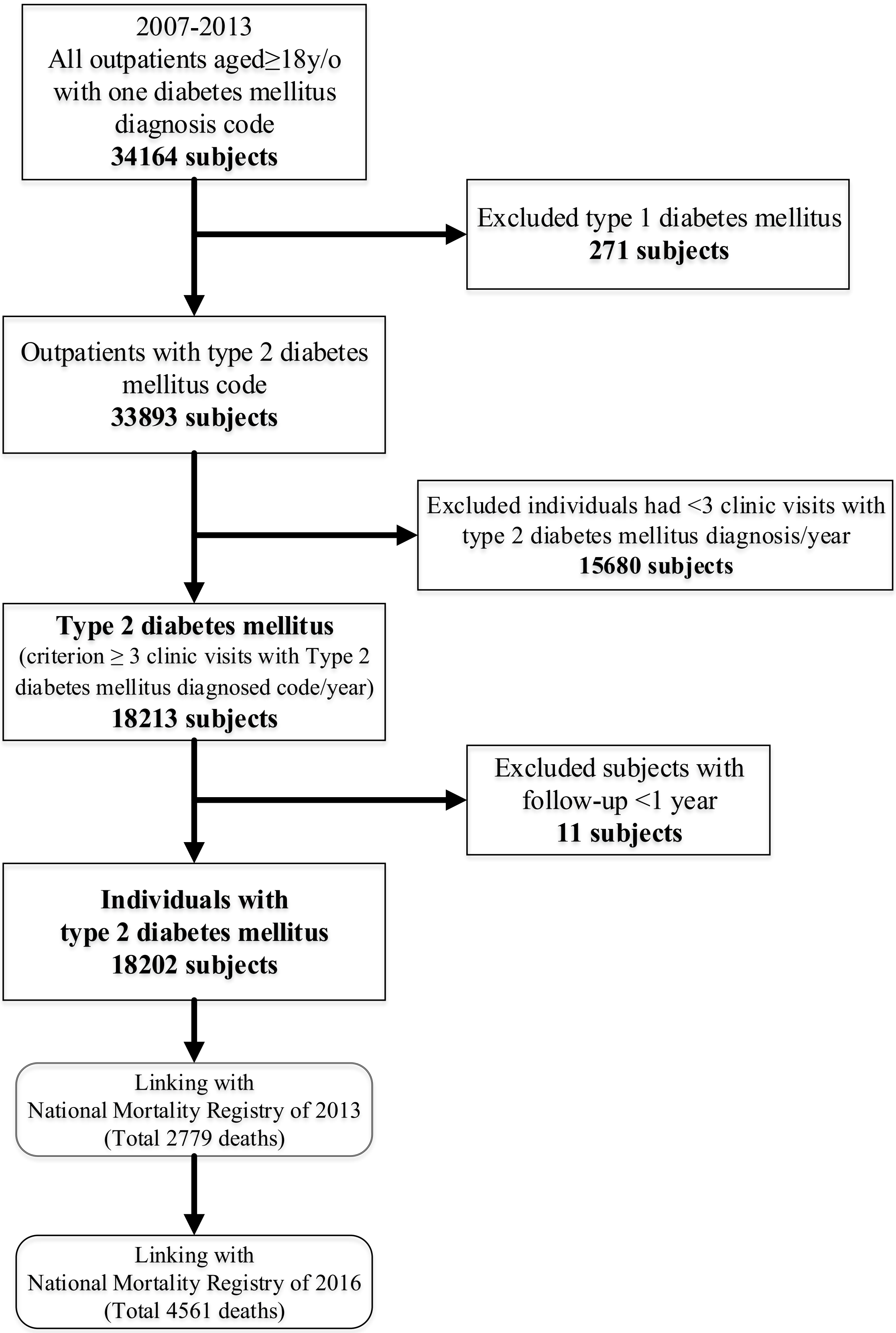

Figure S2. Flow diagram for study subjects

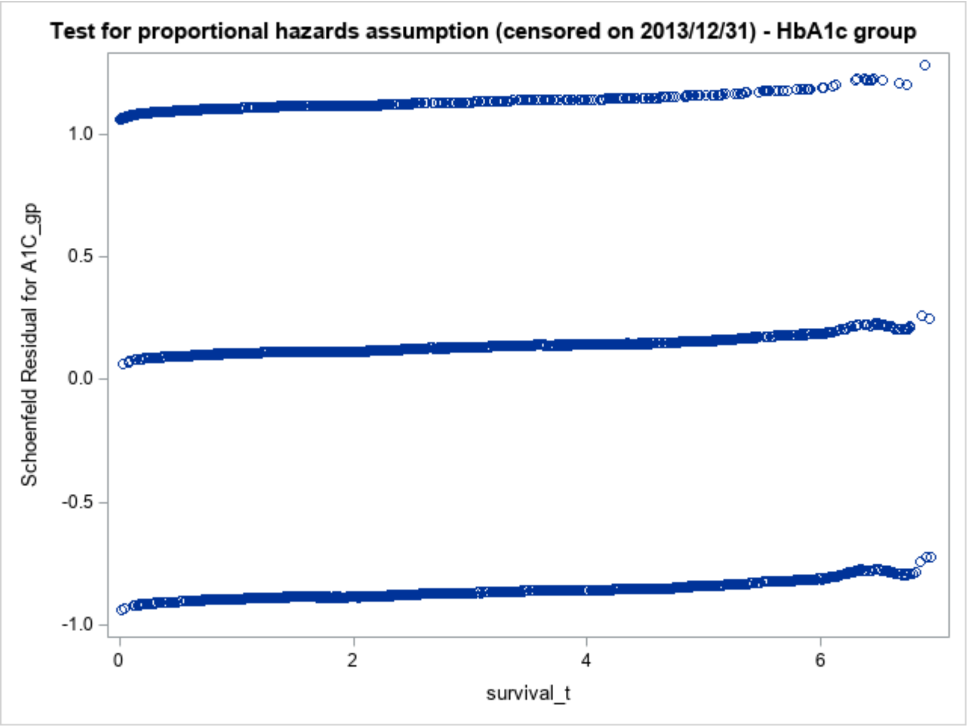

Figure S3(A). Proportional hazards assumption checking for 7-year follow-up

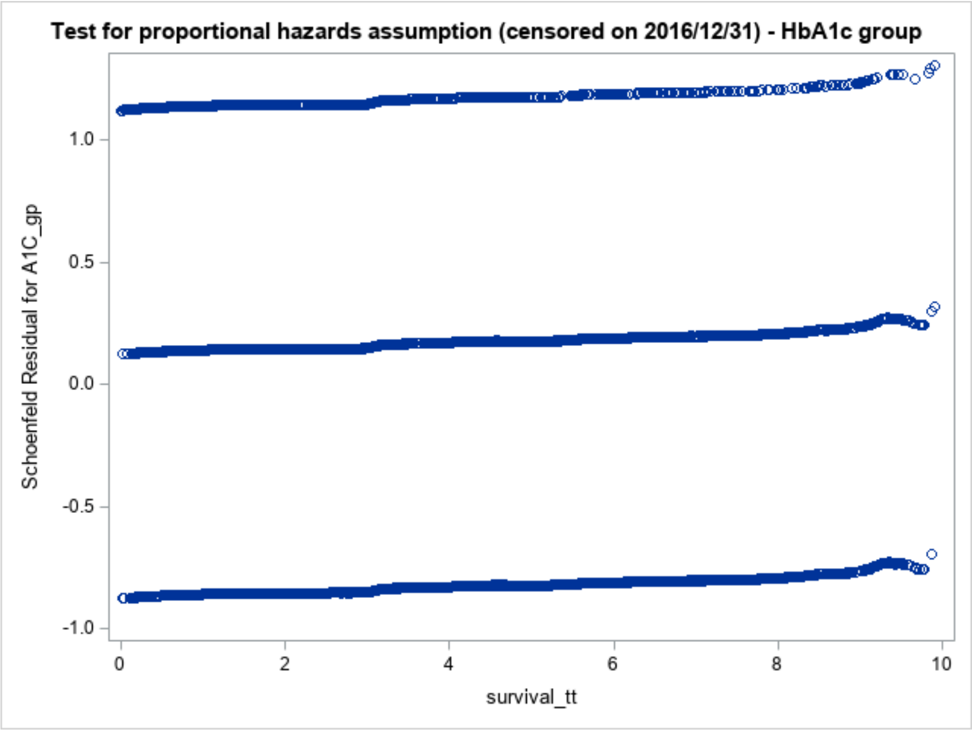

Figure S3(B). Proportional hazards assumption checking for 10-year follow-up

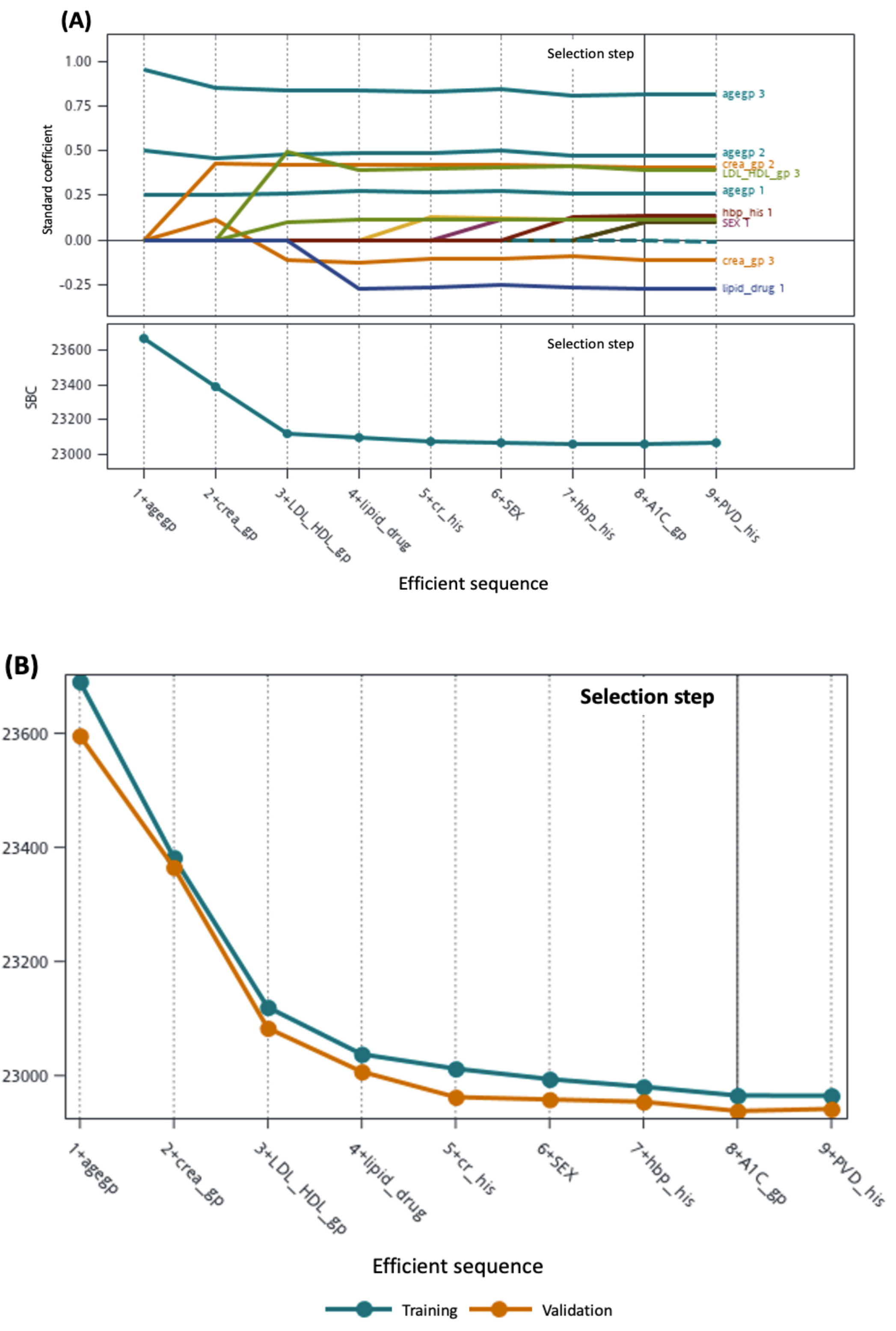

Figure S4. The (A) Coefficient Progression with selection steps and (B) efficient sequence of cross-validation (7-year model)

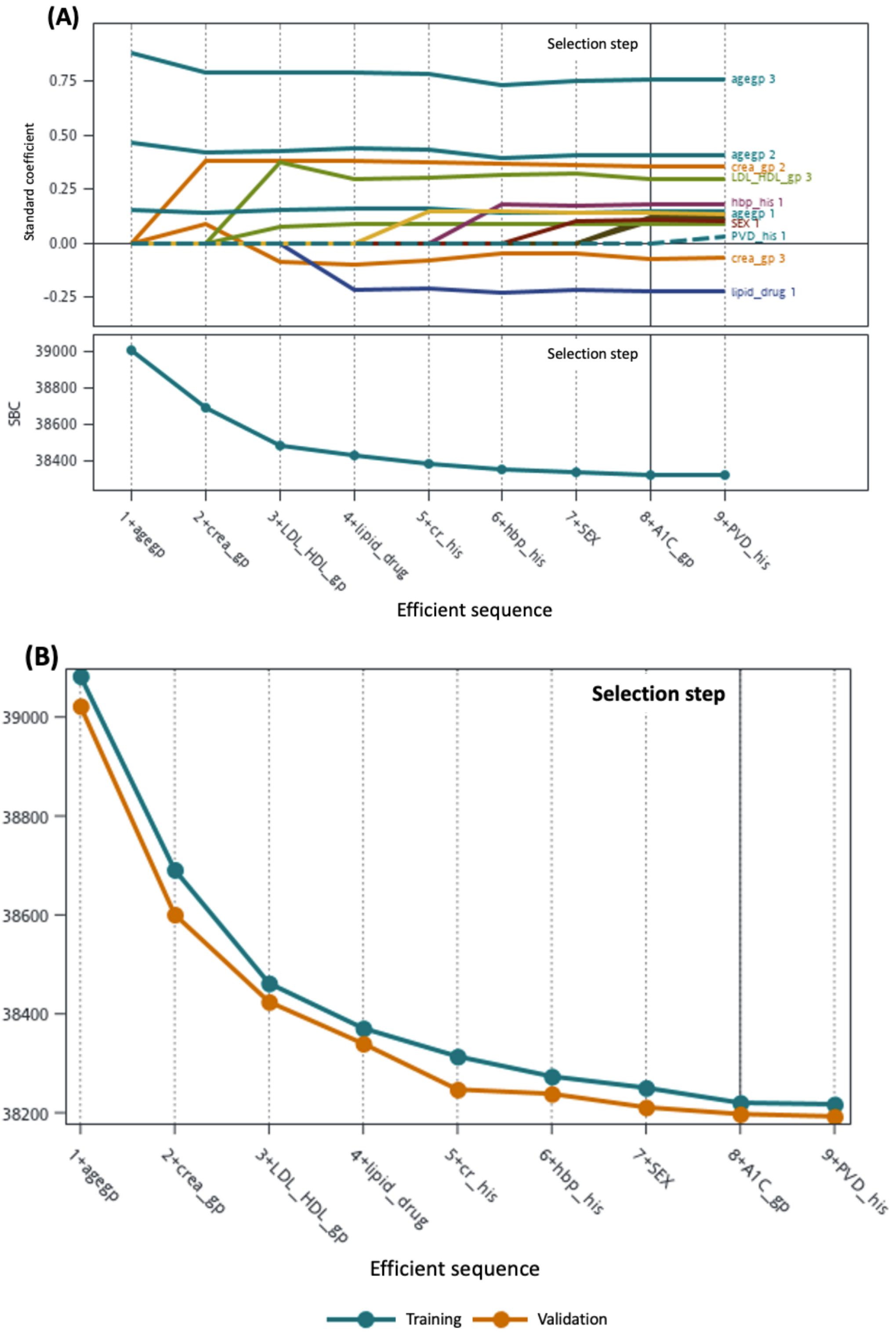

Figure S5. The (A) Coefficient Progression with selection steps and (B) efficient sequence of cross-validation (10-year model)

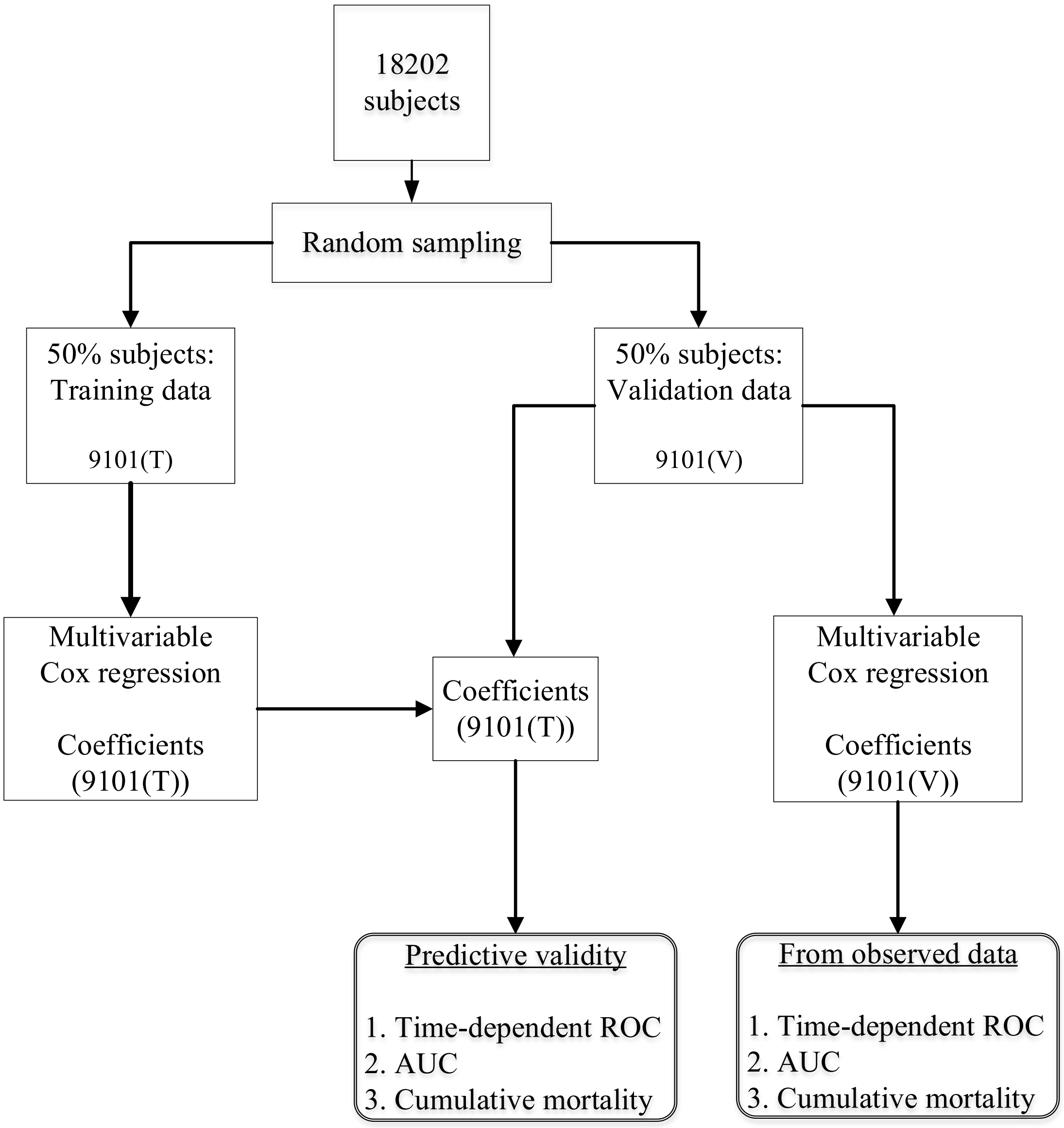

9101(T): training dataset

9101(V): validation dataset

Figure S6. Flowchart for model cross-validation

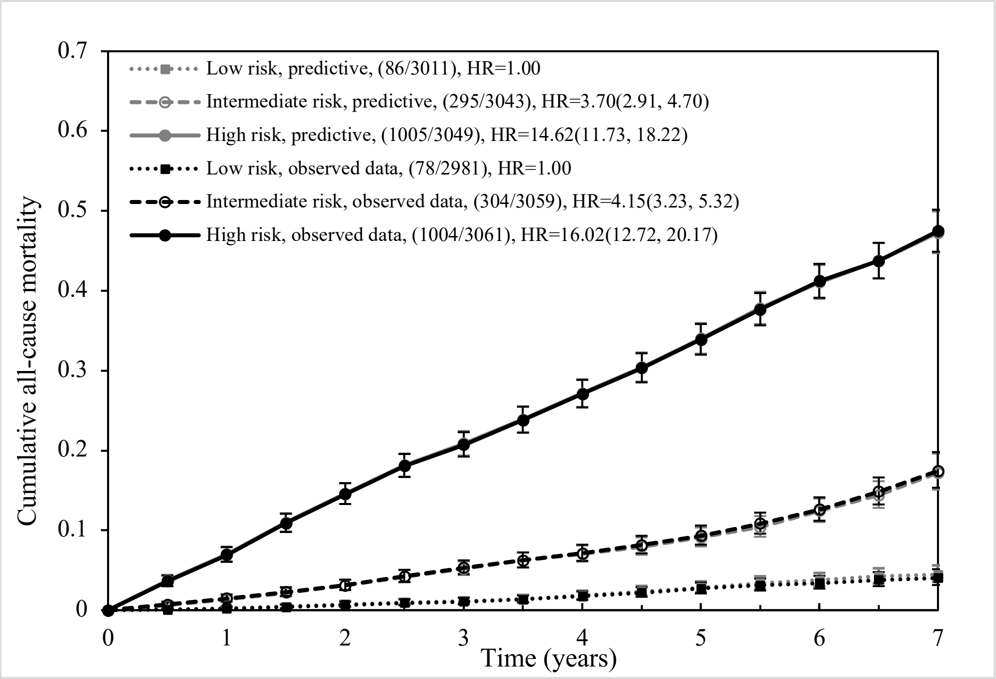

Figure S7(A). Cumulative all-cause mortality for 7-year follow-up based on 9101 validation subjects

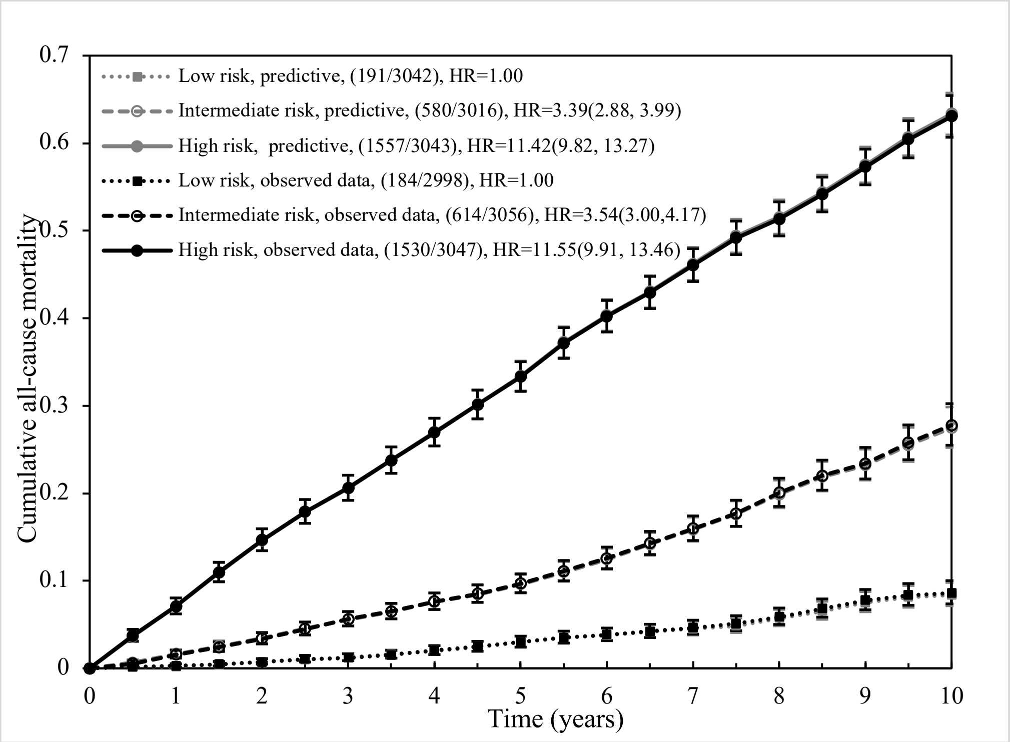

Figure S7(B). Cumulative all-cause mortality for 10-year follow-up based on 9101 validation subjects

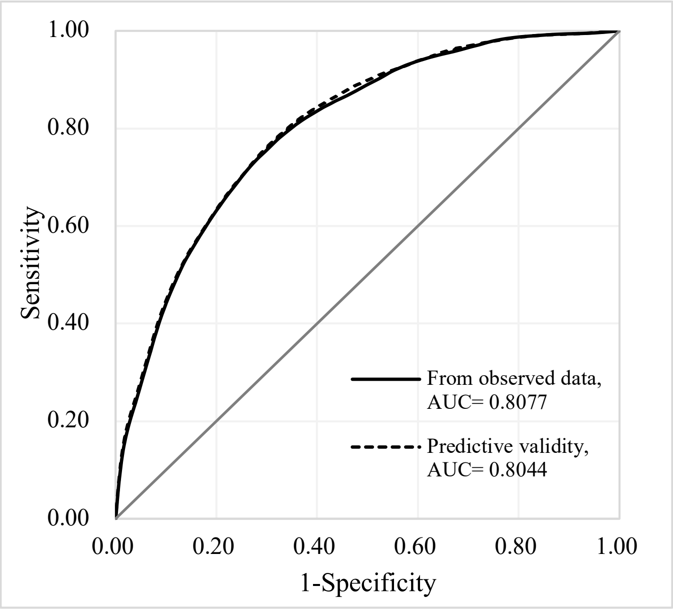

Figure S8(A). ROC and AUC for cross-validation at the 2-year time point based on the 7-year follow-up

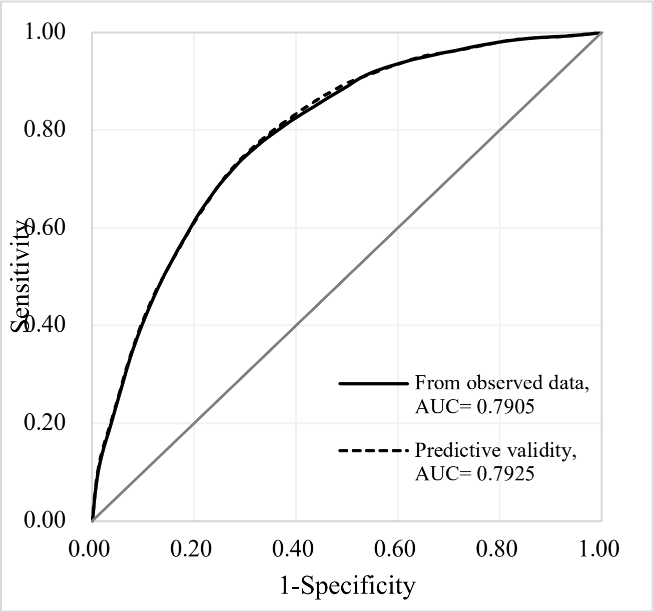

Figure S8(B). ROC and AUC for cross-validation at the 4-year time point based on the 7-year follow-up

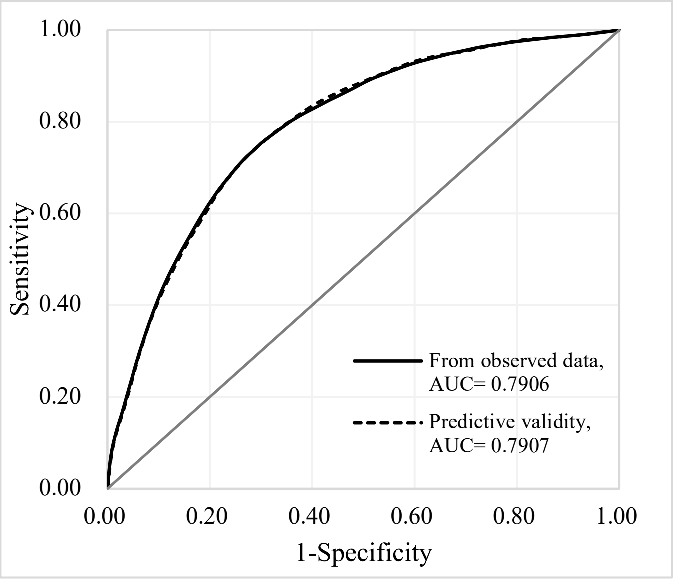

Figure S8(C). ROC and AUC for cross-validation at the 6-year time point based on the 7-year follow-up

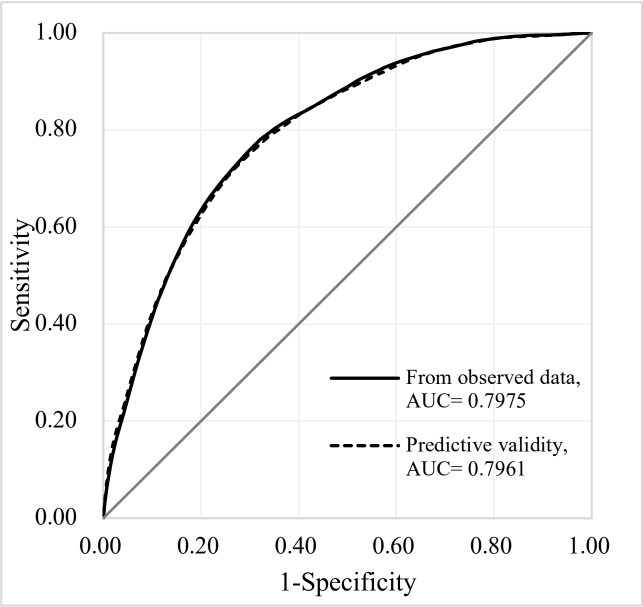

Figure S9(A). ROC and AUC for cross-validation at the 2-year time point based on the 10-year follow-up

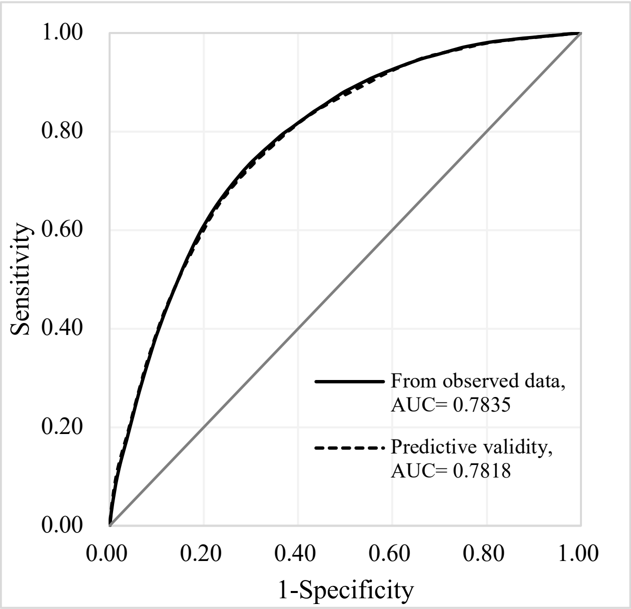

Figure S9(B). ROC and AUC for cross-validation at the 4-year time point based on the 10-year follow-up

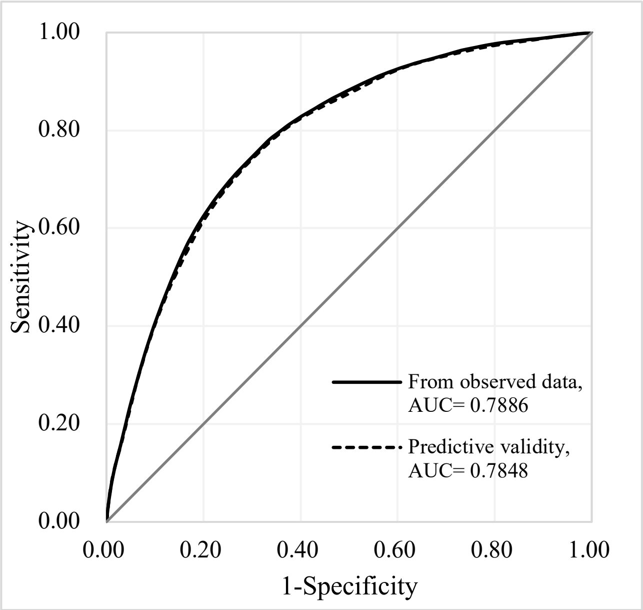

Figure S9(C). ROC and AUC for cross-validation at the 6-year time point based on the 10-year follow-up

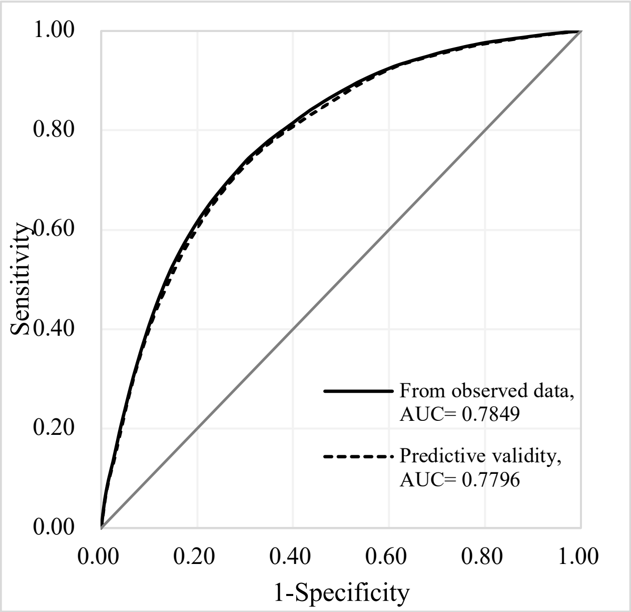

Figure S9(D). ROC and AUC for cross-validation at the 8-year time point based on the 10-year follow-up
