# Supplementary material for: Developing a Prediction Model for 7-Year and 10-Year All-Cause Mortality Risk in Type 2 Diabetes Using a Hospital-Based Prospective Cohort Study
Source: J Clin Med. 2021 Oct 18;10(20):4779. doi: 10.3390/jcm10204779 (PMC8537078; doi:10.3390/jcm10204779)
Supplement: Supplementary file 1 [file jcm-10-04779-s001.zip › 2021-08-25_JCM_revision 2_Supplementary Tables.pdf]

Developing a Prediction Model for 7-year and 10-year All-cause Mortality Risk in Type 2 Diabetes Using a Hospital-based Prospective Cohort Study

Sherry Yueh-Hsia Chiu[1,2], Ying Isabel Chen[3], Jui-fen Rachel Lu[4,5], Soh-Ching Ng[6], Chih-Hung Chen[6*]

1. Department of Health Care Management, College of Management; and Healthy Aging Research Center, Chang Gung University, Taiwan; sherrychiu@mail.cgu.edu.tw (S.Y.-H. Chiu)
2. Division of Hepato-gastroenterology, Department of Internal Medicine, Kaohsiung Chang Gung Memorial Hospital, Taiwan
3. Graduate Institute of Epidemiology and Preventive Medicine, College of Public Health, National Taiwan University, Taipei, Taiwan; glamorous2238@gmail.com (Y.I. Chen)
4. Graduate Institute of Business and Management and Department of Health Care Management, College of Management, Taoyuan, Chang Gung University, Taiwan; rachel@mail.cgu.edu.tw (J.-f. R. Lu)
5. Department of Radiation Oncology, Linkou Chang Gung Memorial Hospital, Linkou, Taiwan
6. Division of Endocrinology and Metabolism, Department of Internal Medicine, Keelung Chang Gung Memorial Hospital, Keelung; Chang Gung University, Taiwan; angelang1127@gmail.com (S.-C. Ng);

   *Correspondence: yh1008@cgmh.org.tw (C.-H. Chen)

Table S1. Description of variables, candidate predictors and definition in this study

Variable	Type	Definition
Flowno	CHAR	De-identified ID
Date_of_entry	Date	YYMMDD10.
Death_2013	CHAR	1: death, 0: alive (follow-up to 2013/12/31)
Death_2016	CHAR	1: death, 0: alive (follow-up to 2016/12/31)
Date_of_death_2013	Date	Date of death (follow-up to 2013/12/31)
Cause of death_2013	CHAR	ICD for cause of death (up to 2013/12/31)
Date_of_death_2016	Date	Date of death (follow-up to 2016/12/31)
Cause of death_2016	CHAR	ICD for cause of death (up to 2016/12/31)
Date_of_end_2013	Date	Date of death (if death_2013=1)<br>2013/12/31 (if death_2013=0)
Date_of_end_2016	Date	Date of death (if death_2016=1)<br>2016/12/31 (if death_2013=0)
Follow_up_time_2013	Numerical	Date of death-Date_of_entry (if death_2013=1)<br>2013/12/31-Date_of_entry (if death_2013=0)
Follow_up_time_2016	Numerical	Date of death (if death_2016=1)<br>2016/12/31-Date_of_entry (if death_2016=0)
Gender	CHAR	1: Male, 0: Female
Baseline_age	Numerical	Age at entry
ICD_code_main		ICD-code for National Health Insurance reimbursement (main code)
ICD_code 1		ICD-code for National Health Insurance reimbursement
ICD_code 2		ICD-code for National Health Insurance reimbursement

Hypertension	CHAR	1: Yes (Diagnosis with ICD and medication of hypertension>=1 times/year),<br>0: No
Hyperlipidemia	CHAR	1: Yes (Diagnosis with ICD and medication of hyperlipidemia>=1 times/year),<br>0: No
Cancer_history	CHAR	1: Diagnosis with ICD of cancer(s), 0: No
PVD	CHAR	1: Diagnosis with ICD PVD>=1 times/year, 0: No
HbA1c	Numerical	Baseline_HbA1c
HbA1c_group	CHAR	Baseline_HbA1c, 1: normal (<7%), 2: abnormal (>=7%), 3: missing
Creatinine	Numerical	Baseline_creatinine
Creatinine_group	CHAR	Baseline_creatinine;<br>1: normal (male 0.64~1.27, female 0.44~1.13 mg/dL), 2: abnormal, 3: missing
Total cholesterol	Numerical	Baseline_total_cholesterol
TC_group	CHAR	Baseline_total_cholesterol; 1: normal (<200mg/dL), 2: abnormal (>=200mg/dL), 3: missing
Triglyceride	Numerical	Baseline_triglyceridel
TG_group	CHAR	Baseline_triglyceridel; 1: normal (<240mg/dL), 2: abnormal (>=240mg/dL), 3: missing
HDL	Numerical	Baseline_HDL
HDL_gp	CHAR	Baseline_HDL; 1: normal (male >40, female>50 mg/dL), 2: abnormal, 3: missing
LDL	Numerical	Baseline_LDL
LDL_group	CHAR	Baseline_LDL; 1: normal (<130mg/dL), 2: abnormal (>=130mg/dL), 3: missing
LDL/HDL ratio	Numerical	Baseline LDL/HDL_ratio
LDL_HDL_Ratio_group	CHAR	Baseline LDL/HDL_ratio;<br>1: normal (male <3.55 %, female<3.22 %), 2: abnormal, 3: missing (if HDL or LDL missing)

Table S2. Distribution of patient characteristics and risk factors by sex

Variables	Female		Male		Overall	
	n	%	n	%	n	%
Year of study entry						
2007	3742	41.28	3761	41.16	7503	41.22
2008	1570	17.32	1381	15.11	2951	16.21
2009	963	10.62	968	10.59	1931	10.61
2010	927	10.23	989	10.82	1916	10.53
2011	598	6.60	663	7.26	1261	6.93
2012	683	7.53	729	7.98	1412	7.76
2013	582	6.42	646	7.07	1228	6.75
Age						
<50	1365	15.06	2221	24.31	3586	19.70
50-59	2234	24.64	2542	27.82	4776	26.24
60-69	2415	26.64	2080	22.76	4495	24.70
≥70	3051	33.66	2294	25.11	5345	29.36
History of cancer						
No	7015	77.39	6923	75.77	13938	76.57
Yes	2050	22.61	2214	24.23	4264	23.43
History of PVD						
No	8785	96.91	8822	96.55	17607	96.73
Yes	280	3.09	315	3.45	595	3.27
History of hypertension						
No	1984	21.89	2077	22.73	4061	22.31
Yes	7081	78.11	7060	77.27	14141	77.69
Use of antihypertensive drugs						
No	2820	31.11	2911	31.86	5731	31.49
Yes	6245	68.89	6226	68.14	12471	68.51
History of hyperlipidemia						
No	2823	31.14	3253	35.60	6076	33.38
Yes	6242	68.86	5884	64.40	12126	66.62
Use of antihyperlipidemic drugs						
No	3863	42.61	4416	48.33	8279	45.48
Yes	5202	57.39	4721	51.67	9923	54.52
HbA1c						
Normal (< 7%)	3630	48.97	3661	49.67	7291	49.32
Abnormal (≧7%)	3782	51.03	3710	50.33	7492	50.68

Missing	1653		1766		3419	
Creatinine						
Normal	5814	72.18	5887	73.19	11701	72.69
Abnormal	2241	27.82	2156	26.81	4397	27.31
Missing	1010		1094		2104	
Total cholesterol						
Normal (< 200 mg/dl)	4656	61.26	5171	68.24	9827	64.75
Abnormal (≧200 mg/dl)	2944	38.74	2407	31.76	5351	35.25
Missing	1465		1559		3024	
Triglyceride						
Normal (< 150 mg/dl)	4799	63.40	4769	63.32	9568	63.36
Abnormal (≧150 mg/dl)	2770	36.60	2763	36.68	5533	36.64
Missing	1496		1605		3101	
LDL						
Normal (< 100 mg/dl)	2568	35.42	2628	36.52	5196	35.97
Abnormal (≧100 mg/dl)	4682	64.58	4568	63.48	9250	64.03
Missing	1815		1941		3756	
HDL						
Normal	1845	25.50	2369	32.85	4214	29.17
Abnormal	5391	74.50	4842	67.15	10233	70.83
Missing	1829		1926		3755	

PVD: peripheral vascular disease;

Normal creatinine level: male <1.27, female:<1.13 mg/dl;

Abnormal HDL level: male<40, female<50 mg/dl.

Table S3. Numbers and causes of deaths by the 7-year and 10-year follow-ups

Causes of death	7-year follow-up		10-year follow-up	
	Deaths	%	Deaths	%
Neoplasm	669	24.1%	1032	22.6%
Iron deficiency anemia/aplastic and other anemias and other bone marrow failure syndromes	2	0.1%	3	0.1%
Diabetes mellitus	459	16.5%	803	17.6%
Motor and/or nerve function assessment/dementia	6	0.2%	22	0.5%
Meningitis	1	0.0%	1	0.0%
Infantile spinal muscular atrophy	14	0.5%	14	0.3%
Parkinson's disease	14	0.5%	19	0.4%
Alzheimer's disease	19	0.7%	19	0.4%
Hypertensive diseases	69	2.5%	116	2.5%
Heart disease (excluding hypertension)	296	10.7%	544	11.9%
Cerebrovascular diseases	176	6.3%	270	5.9%
Aortic aneurysm and dissection	14	0.5%	16	0.4%
Influenza	1	0.0%	6	0.1%
Pneumonia	205	7.4%	370	8.1%
Acute bronchiolitis	9	0.3%	9	0.2%
Chronic lower respiratory diseases	72	2.6%	120	2.6%
Coal worker's pneumoconiosis	16	0.6%	21	0.5%
Lung diseases due to external agents	15	0.5%	22	0.5%
Gastric ulcer/peptic ulcer	3	0.1%	4	0.1%
Hernia	3	0.1%	3	0.1%
Chronic liver diseases/cirrhosis	96	3.5%	148	3.2%
Disorders of gallbladder, biliary tract and pancreas	5	0.2%	8	0.2%
Diseases of the skin and subcutaneous tissue	6	0.2%	13	0.3%
Diseases of the musculoskeletal system and connective tissue	35	1.3%	50	1.1%
Chronic/acute kidney disease	128	4.6%	222	4.9%
Age-related physical debility	3	0.1%	3	0.1%
Injury	56	2.0%	85	1.9%
Self-harm	30	1.1%	51	1.1%
Assault	1	0.0%	2	0.0%
Other	356	12.8%	565	12.4%
Total	2779		4561	

Table S4. AIC for model selection

Follow-up years	Model	Parameters selected	AIC
7-year	A	baseline age, sex, HbA1c, creatinine level, history of cancer, history of PVD, LDL/HDL ratio, history of hypertension	49913.24
	B	baseline age, sex, HbA1c, creatinine level, history of cancer, history of PVD, LDL/HDL ratio, use of anti-hypertensive drugs	49911.84
	C	baseline age, sex, HbA1c, creatinine level, history of cancer, history of hypertension, LDL/HDL ratio, use of anti-hyperlipidemic drugs	**49768.20**
10-year	A	baseline age, sex, HbA1c, creatinine level, history of cancer, history of PVD, LDL/HDL ratio, history of hypertension	82905.47
	B	baseline age, sex, HbA1c, creatinine level, history of cancer, history of PVD, LDL/HDL ratio, use of anti-hypertensive drugs	82885.26
	C	baseline age, sex, HbA1c, creatinine level, history of cancer, history of hypertension, LDL/HDL ratio, use of anti-hyperlipidemic drugs	**82754.09**

AIC: Akaike information criterion

Table S5. Model selection using SBC with best criterion value for 7-year and 10-year model

7-year model					10-year model				
Step	Variable label	Variable	Number effect in	SBC	Step	Variable label	Variable	Number effect in	SBC
1	agegp	Age at entry by group	1	51057.1413	1	agegp	Age at entry by group	1	84254.5753
2	crea_gp	Creatinine level	2	50587.7060	2	crea_gp	Creatinine level	2	83574.2618
3	LDL_HDL_gp	LDL/HDL ratio	3	49993.1190	3	LDL_HDL_gp	LDL/HDL ratio	3	83210.4623
4	lipid_drug	Hyperlipidemia drug use	4	49952.5372	4	cr_his	Cancer history	4	83088.9448
5	cr_his	Cancer history	5	49885.5471	5	lipid_drug	Hyperlipidemia drug use	5	82950.5424
6	A1C_gp	HbA1C level	6	49873.3348	6	SEX	Gender	6	82907.9769
7	SEX	Gender	7	49856.9383	7	hbp_his	Hypertension history	7	82868.8903
8	hbp_his	Hypertension history	8	49846.0562*	8	A1C_gp	HbA1C level	8	82837.9367*
9	PVD_his	PVD history	9	49850.3510	9	PVD_his	PVD history	9	82837.9529

*: best criterion value; SBC: Schwarz Bayesian criterion

Table S6. The results of regression coefficient for 7-year and 10-year using Lasso method

Variable	7-year model	10-year model
	Coefficient	Coefficient
Age at entry		
50-59 vs. <50 y/o	0.2907	0.3014
60-69 vs. <50 y/o	0.8823	0.8989
≥70 vs. <50 y/o	1.6267	1.6301
Sex		
Male vs. Female	0.1734	0.2034
History of cancer		
Yes vs. No	0.3292	0.3784
History of hypertension		
Yes vs. No	0.2515	0.3324
Use of antihyperlipidemic drugs		
Yes vs. No	-0.5304	-0.4196
HbA1c		
≧7 vs. < 7	0.2188	0.1902
Missing vs. < 7	0.1995	0.1960
Creatinine		
Abnormal vs. normal	0.9094	0.8725
Missing vs. normal	-0.2994	-0.1850
LDL/HDL ratio		
Abnormal vs. normal	0.2417	0.1918
Missing vs. normal	0.9003	0.6264

Table S7. Model selection based on training data using SBC with best criterion value for 7-year and 10-year model

**7-year model**					**10-year model**				
Step	Variable label	Variable	Number effect in	SBC	Step	Variable label	Variable	Number effect in	SBC
1	agegp	Age at entry by group	1	23668.3580	1	agegp	Age at entry by group	1	39010.6952
2	crea_gp	Creatinine level	2	23390.2965	2	crea_gp	Creatinine level	2	38692.8811
3	LDL_HDL_gp	LDL/HDL ratio	3	23119.4009	3	LDL_HDL_gp	LDL/HDL ratio	3	38483.0897
4	lipid_drug	Hyperlipidemia drug use	4	23095.1303	4	lipid_drug	Hyperlipidemia drug use	4	38431.7467
5	cr_his	Cancer history	5	23076.2810	5	cr_his	Cancer history	5	38380.5024
6	SEX	Gender	6	23066.2905	6	hbp_his	Hypertension history	6	38353.6455
7	hbp_his	Hypertension history	7	23060.9198	7	SEX	Gender	7	38335.7003
8	A1C_gp	HbA1C level	8	23059.4806*	8	A1C_gp	HbA1C level	8	38321.1542*
9	PVD_his	PVD history	9	23066.3210	9	PVD_his	PVD history	9	38325.5307

*: best criterion value; SBC: Schwarz Bayesian criterion

Table S8. Distribution of patient characteristics and risk factors by training and validation data

Variable		Training data		Validation data		p-value
		n	%	n	%	
**Overall**		**9101**		**9101**		
Mean age		61.6	SD=13.2	61.4	SD=13.3	0.2508
Sex						
	Female	4555	50.0%	4510	49.6%	0.5047
	Male	4546	50.0%	4591	50.4%	
HbA1c						
	Normal	3705	40.7%	3586	39.4%	0.1169
	Abnormal	3730	41.0%	3762	41.3%	
	Missing	1666	18.3%	1753	19.3%	
History of cancer						
	No	6961	76.5%	6977	76.7%	0.7795
	Yes	2140	23.5%	2124	23.3%	
Follow-up outcome (31-Dec-2013)						
	Alive	7708	84.7%	7715	84.8%	0.8853
	Death	1393	15.3%	1386	15.2%	
Follow-up outcome (31-Dec-2016)						
	Alive	6868	75.5%	6773	74.4%	0.1042
	Death	2233	24.5%	2328	25.6%	

Table S9. Harrell's C statistic by sex based on 7-year and 10-year follow-up

Follow-up	Classification	Harrell's C statistic	95% CI
7-year	Overall	0.7955	(0.7873, 0.8037)
	Female	0.8031	(0.7913, 0.8149)
	Male	0.7906	(0.7792, 0.8020)
10-year	Overall	0.7775	(0.7708, 0.7842)
	Female	0.7856	(0.7762, 0.7950)
	Male	0.7709	(0.7615, 0.7803)

CI: confidence interval
